# Assessment of the nutritive value of corn stover and king grass in complete feed on Ongole steer calves productivity

**DOI:** 10.14202/vetworld.2020.801-806

**Published:** 2020-04-28

**Authors:** Ronny Agustinus Victor Tuturoong, Sjenny Sutryaty Malalantang, Sony Arthur Ely Moningkey

**Affiliations:** Department of Animal Feed and Nutrition, Faculty of Animal Science, Sam Ratulangi University, Manado 95115, North Sulawesi, Indonesia

**Keywords:** complete feed, corn stover, king grass, Ongole breed

## Abstract

**Aim:**

This study aimed to assess the nutritional values of corn stover and king grass (*Pennisetum purpupoides*) in complete feed on the productivity of male Ongole steer calves.

**Materials and Methods:**

This study was conducted in two steps: Cattle adaptation and data collection. Cattle adaptation was carried out for 2 weeks, and the cattle were fed the experimental feed. The experimental feed was formulated into five combinations: R1 (50% king grass); R2(12.5% corn stover +7.5% king grass 50% concentrate); R3(25% corn stover+25% king grass); R4(37.5% corn stover+12.5% king grass); and R5(50% corn stover). All experimental feeds were added with 50% concentrate. Data were collected in five intervals, and each interval was of 4weeks. In every interval, weight gain and digestibility were measured every day, beginning from the 4^th^week by collecting feces. Dry matter (DM), organic matter (OM), crude protein (CP) digestibility, neutral detergent fiber (NDF), acid detergent fiber (ADF), body weight gain, and feed conversion data were analyzed.

**Results:**

R4 and R5 treatment significantly increased (p<0.05) the feed nutritional value and weight gain of male Ongole steer calves. Interestingly, treatment with R4 had the maximum increase (p<0.05) on the digestibility value of each variable: DM, 68.85%; OM, 71.89%; CP, 73.90%; NDF, 59.10%; ADF, 55.35%; and weight gain, 0.61/gr/day.

**Conclusion:**

R4 treatment found to be the best nutritional value for improving the productivity of male Ongole steer calves.

## Introduction

Corn stover consists of stalk, leaves, cob, and flower of corn left in the field after harvesting on the 45^th^-46^th^day after their plantation [1]. Corn stover contains slightly more crude protein (CP) (12.06%), crude fiber (CF) (25.02%), and a net energy content of 2350 Kcal/kg [2]. Furthermore, corn stover also contains 91.94% dry matter (DM), 43.23% organic matter (OM), 94% lignin, 4.95% silica, 68.78% neutral detergent fiber (NDF), and 42.36% acid detergent fiber (ADF). The digestibility of DM and OM of corn stover, which ranges from 59.48% to 88.71%, is considerably higher when compared with other fodders [3]. Supplementation with by-products of local crops such as rice bran and legume leaves increases feed intake and digestibility. Corn stover without supplementation has been reported to increases cattle weight by 0.55kg/cow/day, whereas supplementation with rice bran and legume leave increases it by 0.77kg/cow/day [4,5].

Another valuable forage for ruminants is the king grass (*Pennisetum purpupoides*). It possesses high protein content and palatability [6]. King grass has been grown in many locations and has been tested in many laboratories. King grass contains 11.88% CP, 25.48% CF, and a net energy content of 2070 kcal/kg [2], 59.7% of NDF, 0.7% calcium, and 57% total digestible nutrition [7]. King grass has digestibility values in the range of 56.27-87.85%. Ongole is the most favored cattle breed of local farmers in Indonesia. It is a crossbreed between Ongole from India and the local cattle from Java [8]. Ongole breed has long been adapted in Indonesia, especially in North Sulawesi. Ongole breeds are reared to serve dual purposes of beef cattle and working cattle. Ongole breed has also been proven to possess resistance to cow parasites, high temperature, and displays higher tolerance to high-fiber feed [9].

The highest digestibility of DM and OM of corn stover and king grass in the Ongole breed was obtained by a combination of 75% corn stover and 25% king grass that contributed to weight gain up to 3,600,372g/cow/day [3]. However, no data are available for the combination of corn stover and king grass supplemented with concentrated still. Therefore, this study aimed to assess the effect of a combination of corn stover and king grass supplemented with concentrate on Ongole breed productivity.

## Materials and Methods

### Ethical approval

Ethical approval was not required for this study.

### Study design

This study used a 5×5 Latin square design, according to Steel and Torrie [10]. It was carried out in the following stages:

1. Preliminary stage
  Before conducting the study, the animals were placed randomly in the experimental cage. They were adapted to the cage and fed with experimental feed (a combination of corn stover and king grass). Feed adaptation was carried out for 14days. Feeds were given *ad libitum*, and drinking water was provided at all times.
2. The pre-collection stage was carried out 7days before data collection. Afeeding limit of 85% established in the preliminary stage. Feed restriction was performed to maximize feed intake.
3. The collection phase was carried out for 14days for every period (for five periods).
4. The resting period was carried out for 14days. Livestock were given treatment feed to adapt them to the treatment feed and to nullify the effects of the previous period.

Weight gain measurements were taken in every stage, whereas digestibility was measured every day, beginning from the 4^th^week, by collecting the feces. This study aimed to determine feed formulation from a combination of two feed sources (corn stover and king grass) in complete feed. Thus, the treatment (R1-R5) is a comparison/combination of two feed sources, and it does not include controls. In this study, various treatments (combinations) were compared to determine the best formulation.

### Animals and feeding treatment

Twenty-five Ongole male calves aged 26months and weighing 220-245kg were included. Livestock were placed individually in a metallic cage. The effect of corn stover and king grass feed supplemented with concentrate was evaluated by comparing it with complete feed contained isoprotein (13-14%). Proximate analysis of the nutrient content of the feed ingredients used in this study is presented in Table-1, while the formulation and composition of corn stover and king grass feed and complete feed are presented in Table-2.

**Table-1:** Feeding concentration used in this study.

Nutrient	Concentrate (%)	Corn stover (%)	King grass (%)
Dry matter	88.14	23.30	22.20
Protein	17.69	10.55	9.70
Crude fat	10.78	1.90	2.00
Crude fiber	11.25	28.70	36.10
NDF	27.30	63.20	71.10
ADF	14.42	31.30	41.90
Ca	0.73	0.36	0.36
P	1.82	0.21	0.29
Gross energy (Kcal)	3708.89	4346.16	4158.70

NDF=Neutral detergent fiber, ADF=Acid detergent fiber, Ca=Calcium, P=Phosphorus

**Table-2:** Feeding formulation used in this study.

Feed combination	Treatment				

	R1	R2	R3	R4	R5
Corn stover	0	12.5	25	37.5	50
King grass	50	37.5	25	12.5	0
Concentrate	50	50	50	50	50

**Feeding composition**					

Dry matter	55.07	55.23	55.39	55.57	55.72
Organic matter	43.60	44.61	45.63	46.65	47.65
Protein	13.70	13.80	13.91	14.01	14.12
Crude fat	6.39	6.38	6.36	6.35	6.34
Crude fiber	23.68	22.75	21.83	20.90	19.98
NDF	49.20	48.21	47.22	46.24	45.25
ADF	28.16	26.84	25.51	24.19	22.86
Ash	11.47	10.62	9.77	8.92	8.07
Gross energy (Kcal)	2950.35	2967.92	2985.49	3003.07	3020.64

NDF=Neutral detergent fiber, ADF=Acid detergent fiber

### Evaluation of digestibility

The chemical composition of corn stover and king grass was analyzed using a standard procedure for DM according to the AOAC procedure [11]. NDF, ADF, cellulose, hemicellulose, and total lignin were measured according to Van Soest *et al*. [12]. Feed consumption was calculated using the following formula:


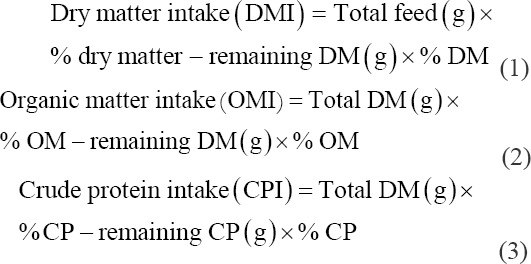


Digestibility of corn stover and king grass was measured according to Tilley and Terry [13]:


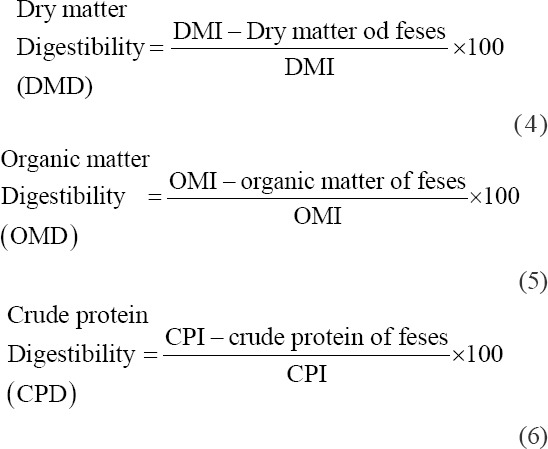


Body weight gain (BWG) was measured, according to Tillman *et al*. [14]. BWG was obtained by subtracting the initial body weight from the weight of the cattle at the end of the treatment, the feed collection period. Daily weight gain was calculated by multiplying BWG by the total number of observations. Metabolic weight was also calculated by weighing feces and urine at the beginning of the collection period.

### Statistical analysis

Data were analyzed using one-way analysis of variance, which performed with the SPSS for Windows (IBM Corp., USA). Significance of difference between all treatment groups was indicated by p<0.05.

## Results

The evaluation of the digestibility value of DM, OM, CF, cellulose, and hemicellulose of corn stover and king grass in complete feed on organochlorine (OC) cattle is presented in Table-3.

**Table-3:** Formulation of feed treatment and composition of feed substances.

Formulation of research feed substance	Treatment				

	R1	R2	R3	R4	R5
Dry matter digestibility (DMD g)	65.95^a^	66.58^a^	66.90^a^	68.85^b^	68.70^b^
Organic matter digestibility (OMD g)	67.98^a^	68.90^a^	70.00^a^	71.89^b^	71.72^b^
Crude protein digestibility (CPD g)	70.90^a^	71.98^a^	73.02^a^	73.90^b^	73.86^b^
NDF digestibility	57.75^a^	57.85^a^	58.15^a^	59.10^b^	59.08^b^
ADF digestibility	51.70^a^	52.65^a^	53.25^b^	55.35^b^	55.20^b^
Body weight gain	0.49^a^	0.53^a^	0.55^a^	0.61^b^	0.59^b^

Different letter in the same row shows significant difference (p≤0.05). NDF=Neutral detergent fiber, ADF=Acid detergent fiber

### DM digestibility (DMD)

The results showed that the DMD of feed comprising of corn stover and king grass in this study ranges from 65.95% to 68.85%. Analysis of variance showed a significant difference (p≤0.05). The highest digestibility was found with R4 treatment (68.85%), which included a combination of feed containing 37.5% corn stover+12.5% king grass+50% concentrate. R5 treatment also showed similar results (DMD– 68.70%), with complete feed containing 50% corn stover, 0% king grass, and 50% concentrate. The DMD values were higher than those obtained with R1, R2, and R3 treatments, which included complete feed containing 0.25-25% corn stover, 25-50% king grass, and 50% concentrate, respectively. The increase in digestibility of DM feed seems proportional to the increasing amounts of corn stover in the complete feed. Data from the proximate analysis (Table-1) showed that the metabolic energy content of corn stover was higher (4346.16 kcal) than that of king grass (4158.70 kcal). The high metabolic energy content of corn stover was due to the presence of readily available carbohydrates (RACs).

### OM digestibility (OMD)

The average OMD of complete feed was observed to be between 67.98% and 71.89%. Analysis of variance showed a significant difference (p≤0.05). The highest average OMD was observed with R4 treatment (71.89%), which was not significantly different from that with R5 treatment (71.72%) but was significantly higher than that with R1, R2, and R3 treatments. The lowest OMD was significant (p=0.05) with R1(67.78%) followed by that with R2 and R3.

### CP digestibility (CPD)

The average CPD in complete feed was observed to be between 70.90% and 73.90%. Analysis of variance showed a significant difference (p≤0.05). R4 and R5 showed no significant difference (p>0.05), but R1, R2, and R3 were significantly different (p<0.05) from R4 and R5. The highest digestibility was observed with R4(73.90%), which included complete feed containing 37.5% corn stover, 12.5% king grass, and 50% concentrate, and the lowest digestibility was observed with R1(70.90%), which included complete feed with 0% corn stover, 50% king grass, and 50% concentrate.

The CPD followed the pattern of DM and OMD. The digestibility of CP in the treatment feed significantly increased with the proportion of corn stover in the complete feed. The increase in DMD was due to the higher metabolic energy content of corn stover compared to that of the king grass. Corn stover has kernels rich in RAC.

### NDF

The average NDF digestibility in this study ranged from 57.75% to 59.10%. Analysis of variance showed that the treatment had a significant effect (p<0.05) on the NDF digestibility in complete feed that included corn stover and king grass. The highest NDF digestibility was found with R4(59.10%), with complete feed containing 37.5% corn stover, 12.5% king grass, and 50% concentrate and was significantly different (p<0.05) as compared to that with R1, R2, and R3, but not with R5. The lowest NDF digestibility was observed with R1(57.74%). The difference in NDF, CP, CF, and energy content between corn stover and king grass in complete feed caused the variability in NDF digestibility. Table-1 shows that the CF content of corn stover is lower (28.70%) as compared to that of king grass (36.10%) and that the NDF content in corn stover (63.20%) is lower than that of king grass (71.10%). In contrast, the CP content in corn stover was higher (10.55%) than that of king grass (9.70%), and the metabolic energy content of corn stover (4346.16 Kcal) is higher than that of king grass (4158.70 Kcal).

### ADF digestibility

ADF digestibility is determined by the microbial population and cellulolytic microbial activity in the rumen. ADF has the same digestibility pattern as NDF because both have almost the same fiber component. ADF consists of cellulose, lignin, and silicates without hemicellulose and only dissolves in acidic solvents. On the other hand, NDF dissolves in neutral solvents and contains hemicellulose, which can be easily digested. In this study, the average ADF digestibility of complete feed that comprised of corn stover and king grass ranged from 51.70% to 55.35% and was lower than NDF digestibility (57.74%-59.10%).

### BWG

The BWG of complete feed comprising of corn stover and king grass for each calf ranged from 0.49 to 0.61/gr/day. Analysis of variance showed a significant difference (p≤0.05). The highest increase was observed with R4(0.61/gr/day). R5 led to a non-significant decrease (0.59/gr/day) and a significant increase was observed with R1, R2, and R3 treatments. The lowest BWG was observed with R1(0.49/gr/day) with a complete feed formulation of 50% king grass and 50% concentrate without the administration of corn stover followed by R2(0.53/gr/day) and R3(0.55/gr/day).

## Discussion

The study showed that the average proportion of young kernels in corn stover was 17%. This readily digested carbohydrate can serve as an energy source for optimal rumen microbial development. The availability of RAC spurs rumen microbial growth so that it has a positive impact on increasing the DMD. In the rumen, the microbial process of degradation of proteins obtained from the feed is faster than the process of providing energy (resulting into formation of ammonium, which can be used as a source of nitrogen), so the presence of corn stover-based RAC increases the efficiency of rumen microbial protein synthesis.

The study conducted by Nasriya *et al*. [3] reported that the average level of corn stover digestibility as a single feed source in OC calves reached 59.48%, and the DMD of king grass as a single feed was 56.27%. Compared with the results of this study, we observe that the digestibility of corn stover as a single forage source with a proportion of 50% in complete feed (R5) is higher (68.70%). On the other hand, the digestibility of king grass as a single forage source, with a proportion of 50% in complete feed (R1) reached 65.95%. The highest digestibility was found in complete feed with a proportion of 37.5% corn stover, 12.5% king grass, and 50% concentrate (R4). The high digestibility of R4 was due to the complementary factor of the combination of corn stover and king grass in complete feed.

The effect of the treatment on the digestibility of feed OM increased in line with the increasing proportion of corn stover in complete feed. The data revealed that the protein and energy content of corn stover were higher than those observed for the king grass. Feed protein is degraded into ammonia by rumen microbes as a nitrogen source, and they also require energy [15] The synthesis rate of rumen microbes was positively correlated with the availability of RAC. We also observed an increase in the digestibility of organic materials. Preston and Leng [16] mentioned that when the protein content in feed is low, it reduces the ammonia concentration in the rumen, resulting in slow growth of rumen microbes and inhibition of carbohydrate degradation.

Protein from feed undergoes degradation into ammonia by rumen microbes as a source of nitrogen for rumen microbes. The degradation rate of protein from feed is determined by the availability of metabolic energy from the feed consumed. The higher the energy obtained from the easily digested carbohydrates, the higher the protein digestibility. CP from feed is degraded by rumen microbes into amino acids and then undergoes deaminase into ammonia as a source of nitrogen and carbon skeleton (α-keto) for rumen microbial growth. The higher CP in the feed consumed, the higher the protein to be degraded to ammonia in the rumen.

Rumen microbes digest NDF more easily than ADF because NDF consists of ADF and hemicellulose, which are easier to be digested by rumen microbes while ADF has no hemicellulose [15]. The level of NDF digestibility in the rumen is affected by the number of fiber-digesting microbial populations in the rumen. Protein from feed is not used directly by rumen microbes but is hydrolyzed by rumen microbes into amino acids and subsequently into ammonia as a source of nitrogen for microbial growth. The utilization effectiveness of protein from feed as a source of nitrogen for microbial growth depends on the availability of energy provided that the carbohydrates are easily digested[17]. The higher the CP and energy and the availability of RAC from feed in the rumen, the higher the rate of growth of the rumen microbial population. The high protein and energy content, as well as the presence of corn kernels as the source of RAC in corn stover compared to king grass, are the reason for the high digestibility of NDF following treatment with R4 and R5. When the CP content in feed is low, the rumen ammonia concentration is low, so rumen microbial growth is slow; consequently, carbohydrate degradation is delayed. Astuti [9] reported that, in regression, there was a relationship between NDF digestibility and CP content in OC calves feed. NDF digestibility is higher in feed with low fiber content than in high feed [16].

The degradation rate of NDF in the rumen was higher than the degradation rate of ADF [18]. The results are shown in Table-3. The highest ADF digestibility was observed with R4(55.35%), complete feed containing 37.5% corn stover, 12.5% king grass, and 50% concentrate, and the lowest was with R1(51.70%), complete feed containing 0% corn bees, 50% king grass, and 50% concentrate. R1 digestibility was not significantly different from R2 and R3, but significantly different from R4 and R5, whereas R4 digestibility was not significantly different (p>0.05) when compared with R5. Astuti [9] reported that the lag time needed by rumen microbes to colonize and initiate digestion of forage in the rumen in NDF was faster (0.7h) than ADF (0.9h), and NDF degradation rate was higher (3.4%/h) compared to ADF (2.9/h). We conclude that the values of ADF are inversely proportional to the digestibility.

The higher average BWG after the treatment of complete feed based on corn stover compared with one being treated with complete feed based on king grass was due to the high digestibility of DM, OM, CP, NDF, and ADF on corn stover-based complete feed[19]. This trend of weight gain was in line with the digestibility levels of DM, OM, CP, NDF, and ADF. The rate of weight gain of cattle is strongly influenced by the digestibility value of the feed consumed. There was a positive correlation between digestibility and weight gain. The level of nutrient digestibility was directly proportional to BWG; the higher the digestibility value of feed, the higher the rate of cattle BWG[15,20].

The increase in BWG was affected by the increase in the proportion of corn stover, which had better nutrient quality than king grass. Acomplementary effect was observed when king grass was added, which then tended to decrease with R5, although not significantly; hence, there was no complementary effect. Data from the proximate analysis (Table-1) show that the metabolic energy content of corn stover is higher (4346.16 kcal) than that of king grass (4158.70 kcal). The proportion of corn kernels from the total weight of corn stover is approximately 17%. Corn kernels contain RAC for optimal rumen microbial growth.

The availability of this RAC spurs rumen microbial growth; therefore, it has a positive impact on increasing the digestibility of feed nutrients [21]. The higher the digestibility of feed nutrients, the higher the feed nutrient is converted to BWG [22]. The CP content of corn stover was 10.55%, which was higher than that of king grass (9.70%). In contrast, the CF of corn stover was 28.78 and that of king grass was 36.10 and ADF of corn stover was 31.30%, which was lower than the ADF of king grass (41.90%). According to Tuturoong [17], the higher the CF and ADF content in the forage feed, the higher the content of lignocellulose, especially lignin, thus the more difficult to digest. The rate of weight gain in cattle is influenced by genetic and environmental factors. The average BWG of each local OC calve from Java and Sumba aged 6months, which was given the king grass *ad libitum* and concentrate of 3kg/day, was 0.51-0.62 kg/day, which was lower than the weight gain of local OC cattle (Javanese and Sumba cattle) crossed with imported cows (Simmental breeds), which was 0.71kg.

## Conclusion

Based on the results of this study, we conclude that the corn waste-and king grass-based feed (37.5% corn waste formulation, 12.5% king grass, and 50% concentrates) provided best nutritional value for local Ongole crossbred calves.

## Authors’ Contributions

RAVT designed and conducted the experiment, acquisition of data, and drafting of the manuscript. SSM conducted the experiment and collected the data. SAEM analyzed the data and drafting the manuscript. All authors read and approved the final manuscript.
